# Development and evaluation of a risk score for type 2 diabetes mellitus among middle-aged Chinese rural population based on the RuralDiab Study

**DOI:** 10.1038/srep42685

**Published:** 2017-02-17

**Authors:** Hao Zhou, Yuqian Li, Xiaotian Liu, Fei Xu, Linlin Li, Kaili Yang, Xinling Qian, Ruihua Liu, Ronghai Bie, Chongjian Wang

**Affiliations:** 1 Department of Epidemiology and Biostatistics, College of Public Health, Zhengzhou University, Zhengzhou, Henan, P.R. China; 2 Department of Clinical Pharmacology, School of Pharmaceutical Science, Zhengzhou University, Zhengzhou, Henan, P.R. China

## Abstract

The purpose of this study was to establish a simple and effective risk score for type 2 diabetes mellitus (T2DM) in middle-aged rural Chinese. Total of 5453 participants aged 30–59 years from the Rural Diabetes, Obesity and Lifestyle (RuralDiab) study were recruited for establishing the RuralDiab risk score by using logistic regression analysis. The RuralDiab risk score was validated in a prospective study from Henan Province of China, and compared with previous risk scores by using the receiver-operating characteristics cure. Ultimately, sex, age, family history of diabetes, physical activity, waist circumference, history of dyslipidemia, diastolic blood pressure, body mass index were included in the RuralDiab risk score (range from 0 to 36), and the optimal cutoff value was 17 with 67.9% sensitivity and 67.8% specificity. The area under the cures (AUC) of the RuralDiab risk score was 0.723(95%*CI*: 0.710–0.735) for T2DM in validation population, which was significant higher than the American Diabetes Association score (AUC: 0.636), the Inter99 score (AUC: 0.669), the Oman risk score (AUC: 0.675). The RuralDiab risk score was established and demonstrated an appropriate performance for predicting T2DM in middle-aged Chinese rural population. Further studies for validation should be implemented in different populations.

With rapid economic development, life-style of human beings has been changed dramatically globally. The prevalence and incidence of type 2 diabetes mellitus (T2DM) are increasing at fast speed in the world. Issued in 2015, the International Diabetes Federation (IDF) estimated that the number of global diabetes individuals aged 20–79 was 415 million and will increase to 642 million by 2040^1^. In China, the prevalence of T2DM was 11.6% in 2010^2^. Similarly developing trend of T2DM in rural areas of China has been sharply increased from less than 1% in 1980 to 10.3% in 2010^2^,^3^. Although the development of urbanization is speeding up in the recent years, the Chinese rural population is still very large. Up to 2015, 44% population lives in rural area of China. Therefore, the prevention and control of T2DM are urgent in rural areas. In addition, diabetes is a major risk factor of cardiovascular diseases including ischemic heart disease and stroke, which accounted for an estimated 12.9 million death globally in 2010^4^,^5^. Screening high risk individuals, taking effectively preventive measures, and avoiding from the risk factors of T2DM are good strategies in prevention and delay of T2DM occurring and its cardiovascular complications.

Personalized intervention is helpful to prevent or delay T2DM by life-style changed and pharmaceutical interfering^6^. Fasting plasma glucose (FPG), oral glucose tolerance test (OGTT) and HBA1c are commonly used for T2DM determination in clinical and epidemiological studies^7^. However, their application has some limitations, which can’t succinctly identify high risk individuals and screen large population on spot. Many risk factors associated with diabetes can be used to recognize high-risk individuals for early intervention^8^,^9^. Risk scores based on some risk factors without laboratory tests have been demonstrated as an effective, low cost and noninvasive tool for identifying the high-risk individuals of T2DM^10^,^11^,^12^,^13^,^14^. Because of incomplete health care system and underdeveloped economy in rural areas, the prevalence of T2DM is already high and continuously increasing in rural areas of China^2^,^3^. Thus, establishing a suitable risk score must be useful in identifying high risk individuals for the prevention and control of T2DM in rural areas.

A risk score of T2DM had been developed according to the data of a nationwide study in China^14^. However, because of quickly increased prevalence of T2DM and the different levels of risk factors in rural population of China, we tried to establish a rural risk assessment tool (the RuralDiab risk score) for T2DM based on the data from the Rural Diabetes, Obesity and Lifestyle (RuralDiab) study. Another prospective study from Henan Province was used to validate and compare the performance between the RuralDiab risk score and previous risk scores.

## Results

### Population characteristics

The characteristics of establishment population was shown in Table 1, which showed that the crude prevalence of undiagnosed T2DM was 4.29% (234 of 5453 individuals), while age, marital status, family history of diabetes, more vegetable and fruit intake, treated with anti-hypertensive medication and body mass index (BMI) had no sex difference. The percentages of high fat intake, current smoking, hypertension and dyslipidemia were higher, but physical activity was lower in men than that in women. Detailed characteristics of validation population were presented in Supplementary Table 1. A total of 249 patients of T2DM were detected in the validation population with a 6-year follow-up.

### Establishment of risk score

Table 2 describes the results of the multivariate logistic regression analysis. The characteristics of establishment population were significantly associated with undiagnosed T2DM included sex, age, family history of diabetes, physical activity, waist circumference, history of dyslipidemia, diastolic blood pressure (DBP), and BMI. The well-fitting was shown by Hosmer-Lemeshow test (*χ*^2^ = 5.25, *P* = 0.731), which the observed prevalence matched well with the predicted prevalence of undiagnosed T2DM in the multivariate logistic regression model. BMI, DBP and history of dyslipidemia by the net reclassification improvement (NRI) analysis were added in the multivariate logistic regression model fitting with sex, age, family history of diabetes, physical activity and waist circumference. The results of analysis showed that the contribution of DBP is higher compared with systolic blood pressure (SBP) for risk score of T2DM, and the strength co-linearity was found between DBP and SBP for effecting on T2DM. In the sensitivity analysis of predicting T2DM, the area under the curve (AUC) of DBP was bigger than that of SBP in the multivariate logistic regression model. Thus, the DBP was incorporated into the risk score of the T2DM. And they improved the predicted probabilities with NRI = 0.2192 (*Z* = 4.67, *P* < 0.001). Detail of NRI was presented in Supplementary Table 2. The AUC of model with BMI, DBP and history of dyslipidemia (AUC = 0.718) was significantly higher than that of the model without BMI, DBP and history of dyslipidemia (AUC = 0.684) (*P* = 0.010) in establishment population. A simple score was derived from the coefficients (*β*) of logistic regression model: *β* < 0.3 was one, 0.3 ≤ *β* < 0.6 was two, 0.6 ≤ *β* < 0.9 was three, 0.9 ≤ *β* < 1.2 was four, 1.2 ≤ *β* < 1.5 was five, 1.5 ≤ *β* < 1.8 was six, 1.8 ≤ *β* < 2.1 was seven, 2.1 ≤ *β* < 2.4 was eight. Finally, the RuralDiab risk score was established with range from 0 to 36.

### Validation the RuralDiab risk score and its advantages compared with others

Table 3 presents the validation of the RuralDiab risk score for predicting risk of T2DM in an external prospective study. The AUCs of the RuralDiab risk score were 0.723 (95% *CI*: 0.710–0.735) in total population, 0.711 (95% *CI*: 0.688–0.732) in men and 0.726 (95% *CI*: 0.709–0.742) in women. The optimal cutoff value was 17 in total population. The AUCs of the RuralDiab risk score was better than that of the American Diabetes Association (ADA) score (AUC: 0.636 in total, 0.628 in men), the Inter99 score (AUC: 0.669 in total, 0.618 in men), the Oman risk score (AUC: 0.675 in total, 0.659 in men) in total population and men. The significant difference of the AUC was only found between the RuralDiab risk score and the ADA score in women (AUC: 0.648). Comparing with the New Chinese Diabetes risk score, the RuralDiab risk score significantly improved the reclassification in all risk scores, and the net reclassification improvement (NRI) were 6.33% in total, 3.86% in men, 9.23% in women, respectively.

**Figure 1:**
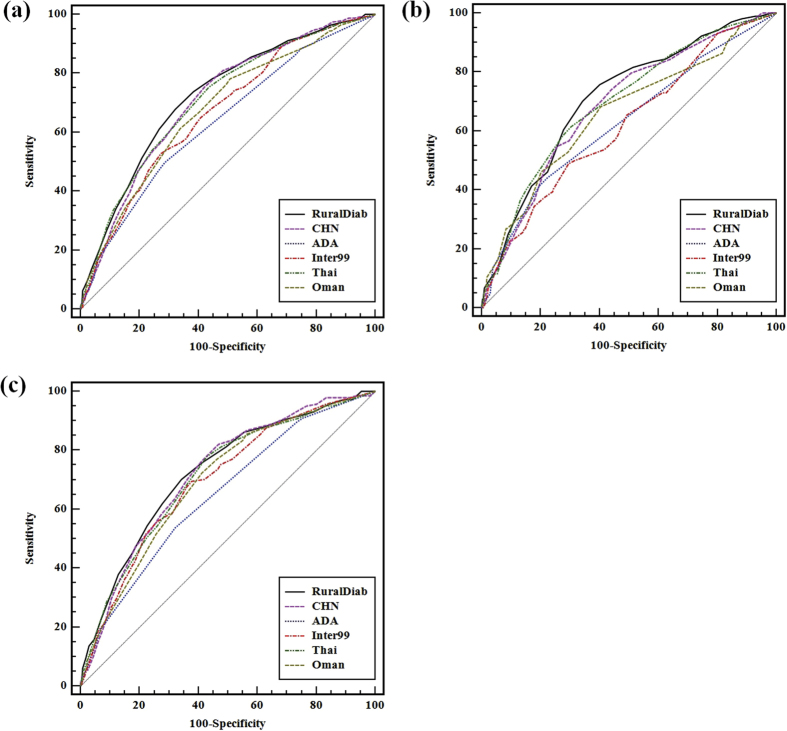
The comparison of the AUC for different risk scores to predict T2DM in validation population ((**a**) Total, (**b**) Men, (**c**) Women). RuralDiab = the RuralDiab risk score; CHN = the New Chinese Diabetes Risk Score; ADA = the American Diabetes Association score; Inter99 = the Inter99 score; Thai = the Thai risk score; Oman = the Oman risk score; T2DM = type 2 diabetes mellitus; AUC = area under the curve.

The Figure 1 showed that the comparison between the RuralDiab risk score and previous risk scores was executed.

## Discussion

The RuralDiab risk score, which was developed from a large-scale rural population study, is the first risk assessment tool for T2DM with noninvasive factors in rural population. Meanwhile, the RuralDiab risk score was validated and evaluated by an external prospective study for T2DM prediction, which showed some advantages of the RuralDiab risk score compared with previous risk scores.

The result of Framing-ham Offspring Study reported that the incident of T2DM was mainly in middle-aged adults^15^. Therefore, the RuralDiab risk score was established in Chinese with aged 30–59 years living in rural area. Previous reports showed that T2DM was a multi-factor metabolic disorder disease, and environment factors and life-style played important roles^16^,^17^,^18^,^19^,^20^,^21^. The results of data analysis found that sex, age, family history of diabetes, physical activity, waist circumference, history of dyslipidemia, DBP, BMI were included in the RuralDiab risk score. Compared with previously published the New Chinese Diabetes Risk Score, the RuralDiab risk score added physical activity and history of dyslipidemia, and made DBP substituted for SBP with adjusting “treated with anti-hypertensive medication”.

With some advantages compared with previous risk scores, especially in validity of T2DM risk prediction, the RuralDiab risk score is a reliable and inexpensive health check tool, which could be used for screening diabetes in the large population. Although it might inevitably omit individuals with T2DM risk^22^,^23^, there are some clinical meanings. Firstly, applying the RuralDiab risk score to predict T2DM may reduce the suffering of individuals with invasive procedure. Secondly, the application of the RuralDiab risk score could quickly identify the high-risk individuals of T2DM in rural areas for both the general population and health care providers. Finally, wide application of the RuralDiab risk score could improve the public awareness of T2DM and help people realize the relevant risk factors.

Although the RuralDiab risk score is the first rural assessment tool for T2DM in China based on a large-scale, population-based data— the RuralDiab study, there are some limitations. Firstly, the cases of undiagnosed T2DM were ascertained by fasting glucose level without OGTT or HBA1c, which might omit some potential T2DM individuals, and OGTT or HBA1c will be considered in future study. Secondly, some important covariates, such as dietary and lifestyle might have reporting bias, but potential covariates were adjusted as much as possible. Thirdly, the current performance might be not ideal enough for risk prediction in practice, and some new indicators or biomarkers, especially for hereditary factors could improve the performance of the risk score in the future. Finally, only one provincial data was applied to establish and validate the RuralDiab risk score, which might limit the popularization and application. In addition, the performance of the risk tool need to be further confirmed in the multi-centered prospective studies.

In conclusion, the current study develops the RuralDiab risk score including sex, age, family history of diabetes, physical activity, waist circumference, history of dyslipidemia, DBP and BMI for predicting T2DM. Compared with the previously published risk scores, the RuralDiab risk score was more suitable for rural population, which might be helpful for rural health care practitioners to assess the risk of T2DM, and then improve the awareness of disease prevention for rural population. However, the potential clinical application remains to be determined.

## Methods

### Study design and participants

Establishment population of the RuralDiab risk score was derived from the Rural Diabetes, Obesity and Lifestyle (RuralDiab) study. In brief, the participants were selected by stratified random cluster sampling from eligible candidates listed in the residential registration record. Firstly, 3 townships were selected from 22 rural areas of Yuzhou County in consideration of the adherence and local medical conditions. Secondly, all permanent residents who satisfied the inclusion criteria and signed informed consent were selected as the subjects. Ultimately, a total of 11032 participants aged 18 years and older were recruited between July and August in 2015 from Yuzhou County in Henan Province of China. The participants were excluded based on the criteria, which comprised (1) previously diagnosed diabetes (n = 818); (2) aged younger than 30 or older than 59 years (n = 4725); (3) with incomplete information (n = 36). Finally, the information of 5453 participants aged 30–59 years was used to establish the RuralDiab risk score of T2DM in the present study.

An external population from one prospective study was used as validation population to evaluate the RuralDiab risk score. The baseline study was conducted from 2007 to 2008, and 10009 participants aged 18 years and above who lived in their current location with at least 10 years were recruited from Xinan County in Henan Province of China. Then, participants were followed up during 2013 and 2014. Individuals with the drop-off (n = 1280), the death (n = 580), age younger than 30 or older than 59 years (n = 1627), diagnosed diabetes at baseline (n = 654), and incomplete information at baseline or follow-up (n = 1215) were excluded. Ultimately, 4653 participants aged 30 to 59 years were included in the current study.

The two surveys were approved by the Zhengzhou University Medical Ethics Committee, and written informed consent was obtained from all participants. The studies were executed with the principles of the Declaration of Helsinki.

### Data collection and laboratory measurement

Using standardized methods for stringent levels of quality control, a standard questionnaire was given to each participant with face-to-face interview by well trained public health workers and physicians to collect information on demographics (age, sex, income status, educational level and marital status), family and individual disease history (diabetes, hypertension, coronary heart disease and stroke), dietary intake and lifestyle (smoking, alcohol drinking, intakes of fat, vegetable and fruit, and physical activity). Age was classified into three categories: ≥30 and <40, ≥40 and <50, ≥50 and <60 years. The educational level was classified into four categories: illiterate, primary school, secondary school, and college and above. Marital status was classified into two categories: married/cohabitation and unmarried/divorced/widowed. Family history was defined as the parents or siblings of participants with a history of disease.

Food frequency method was used to estimate the daily intake of fat, vegetable and fruit in the past one year according to the China Food Composition Table^24^. Based on the Chinese Dietary Guidelines, the appropriate consumption of vegetable and fruit should be more than 500 g daily, and high fat intake was defined as consuming an average of more than 75 g per day^25^. Physical activity for each participant was classified as low, moderate and high level based on the International Physical Activity Questionnaire (IPAQ)^26^. The participants with high or/and moderate level of physical activity were defined as physical activity. Smoking status was classified as current smoking and not current smoking. Participants who were current smoking at least one cigarette per day along with sequential or cumulative 6 months were defined as current smoking according to the definition of the World Health Organization^27^.

Followed the standard procedure, body weight, waist circumference and height of the participants were measured twice to the nearest 0.1 kg and 0.1 cm respectively, and the average values were taken. Blood pressure and heart rate were measured in the sitting position by a standardized protocol^28^. Waist circumference (in centimeter) was classified into five categories: <80, ≥80 but <90, ≥90 but <100, ≥100 but <110, ≥110 in men and <70, ≥70 but <80, ≥80 but <90, ≥90 but <100, ≥100 in women. Body mass index (as kg/m^2^) was classified into six categories: <22, ≥22 but <24, ≥24 but <28, ≥28 but <30, ≥30 but <32, ≥32. Diastolic blood pressure (in mmHg) was classified into four categories: <70, ≥70 but <80, ≥80 but <90, ≥90 or treated with anti-hypertensive medication.

Blood specimens were collected with vacuum tubes containing ethylene diamine tetraacetic acid (EDTA)-K2 after overnight fasting and were centrifuged at 4 °C and 3000 rpm for 10 min. The plasma was transferred with the cold chain and stored at −80 °C for biochemical analyses. Plasma glucose was measured using a modified hexokinase enzymatic method.

### Definitions

Undiagnosed T2DM was defined as having fasting plasma glucose level ≥7.0 mmol/L without previously diagnosed diabetes based on the American Diabetes Association (ADA) diagnostic criteria^29^. After excluding type 1 diabetes mellitus, gestational diabetes mellitus, and other special type diabetes, T2DM was defined as a self-reported diagnosed diabetes or undiagnosed T2DM. All participants brought their prescribed medications during the investigation, and a self-reported history of diabetes was confirmed by the use of insulin or oral hypoglycemic agents. In addition, the hospitalized patients with diabetes had their charts reviewed.

### Previous risk scores selection

This study selected previously representative risk scores of T2DM with noninvasive measures in varied regions and ethnicity, including the American Diabetes Association score (ADA)^10^ from Americans, the Inter99 score (Inter99)^11^ from Europeans, the Thai risk score (Thai)^12^ from Thais, the Oman risk score (Oman)^13^ from Arabians and the New Chinese Diabetes Risk Score (CHN)^14^ from Chinese to compare with the RuralDiab risk score (RuralDiab) in validation population.

### Statistical analysis

Data of the participants’ characteristics were compared. The categorical variables and continuous variables were analyzed through *Chi*-square and *t*-test, respectively. In this analysis, we re-categorized these parameters and used logistic regression analysis to select factors and derive the risk score. Forward stepwise likelihood ratio method of multivariate logistic regression analysis was used to investigate significant risk factors for the RuralDiab risk score. Net reclassification improvement analysis was used to identify whether adding some risk factors could improve the classification of the predicted probabilities of the multivariate logistic regression model^30^. The quintiles of predicted probabilities of having diabetes according to the model comprised of sex, age, family history of diabetes, physical activity, waist circumference were classified into five categories: ≤2.1%, >2.1% and ≤2.9%, >2.9% and ≤3.9%, >3.9% and ≤5.8%, and >5.8%. The risk score was calculated according to the coefficients (*β*) of the model. Then, the receiver-operating characteristics curves were plotted for the RuralDiab risk score, the sensitivity was plotted on the *y*-axis, and the false-positive rate (1-specificity) was plotted on the *x*-axis. The area under the curves reflected the discriminating accuracy of different curves using different combinations of predictors^31^, and the optimal cutoff point was the peak of the curve. Sensitivity, specificity, likelihood ratio, predictive value and the AUC were applied to compare the performance among different risk scores.

A two-tailed *P*-value < 0.05 was deemed statistically significant. Statistical analyses were performed using SAS 9.3 (SAS Institute, USA).

## Additional Information

**How to cite this article**: Zhou, H. *et al*. Development and evaluation of a risk score for type 2 diabetes mellitus among middle-aged Chinese rural population based on the RuralDiab Study. *Sci. Rep.* **7**, 42685; doi: 10.1038/srep42685 (2017).

**Publisher's note:** Springer Nature remains neutral with regard to jurisdictional claims in published maps and institutional affiliations.

## Supplementary Material

Supplementary File

## Figures and Tables

**Table 1:** Population characteristics of establishment population from the RuralDiab study for developing the RuralDiab risk score.

Characteristics	Men(n = 1746)	Women(n = 3707)	Total(n = 5453)	*P*-value
Age (years, mean ± SD)	47.92 ± 6.97	48.30 ± 6.71	48.18 ± 6.80	0.053
Education, n (%)				<0.001
Illiterate	23 (1.32)	160 (4.32)	183 (3.36)	
Primary school	231 (13.23)	765 (20.64)	996 (18.27)	
Secondary school	1358 (77.78)	2649 (71.46)	4007 (73.48)	
College and above	134 (7.67)	133 (3.59)	267 (4.90)	
Marital status, n (%)				0.196
Married/cohabitation	1681 (96.28)	3541 (95.52)	5222 (95.76)	
Unmarried/divorced/widowed	65 (3.72)	166 (4.48)	231 (4.24)	
Family history of diabetes, n (%)	119 (6.82)	309 (8.34)	428 (7.85)	0.052
High fat intake, n (%)	717 (41.07)	924 (24.93)	1641 (30.09)	<0.001
More vegetable and fruit intake, n (%)	479 (27.43)	977 (26.36)	1456 (26.70)	0.401
Current smoking, n (%)	921 (52.75)	7 (0.19)	928 (17.02)	<0.001
Treated with anti-hypertensive medication, n (%)	181 (10.37)	369 (9.95)	550 (10.09)	0.637
Physical activity, n (%)	1104 (63.23)	2628 (70.89)	3732 (68.44)	<0.001
Waist circumference (cm, mean ± SD)	88.24 ± 9.94	84.07 ± 9.36	85.40 ± 9.75	<0.001
Body mass index (kg/m^2^, mean ± SD)	25.41 ± 3.37	25.57 ± 3.42	25.52 ± 3.40	0.113
Systolic blood pressure (mmHg, mean ± SD)	124.31 ± 16.45	119.85 ± 18.22	121.28 ± 17.80	<0.001
Diastolic blood pressure (mmHg, mean ± SD)	81.17 ± 12.00	77.29 ± 11.35	78.53 ± 11.70	<0.001
Hypertension, n (%)	518 (29.67)	854 (23.04)	1372 (25.16)	<0.001
Dyslipidemia, n (%)	772 (44.22)	970 (26.17)	1742 (31.95)	<0.001
Fasting glucose (mmol/L, mean ± SD)	5.47 ± 1.29	5.32 ± 1.11	5.37 ± 1.18	<0.001
Triglycerides (mmol/L, mean ± SD)	1.90 ± 1.39	1.58 ± 1.10	1.68 ± 1.21	<0.001
HDL-C(mmol/L, mean ± SD)	1.15 ± 0.36	1.27 ± 0.45	1.23 ± 0.43	<0.001
Undiagnosed T2DM, n (%)	97 (5.56)	137 (3.70)	234 (4.29)	0.002

SD = standard deviation; HDL-C = high density lipoprotein cholesterol.

**Table 2:** Logistic regression model with undiagnosed T2DM for the RuralDiab risk score in the establishment population.

Risk factors	*OR*(95%*CI*)	*β*-coefficient	Score
Sex			
Women	1.00	—	0
Men	2.12 (1.59–2.82)	0.749	3
Age, years			
30~	1.00	—	0
40~	2.33 (1.19–4.55)	0.845	3
50~	3.89 (2.02–7.47)	1.357	5
Family history of diabetes			
No	1.00	—	0
Yes	1.93 (1.28–2.91)	0.656	3
Physical activity			
Yes	1.00	—	0
No	1.42 (1.08–1.87)	0.352	2
Waist circumference, cm			
<80 (men) or <70 (women)	1.00	—	0
80~ (men) or 70~ (women)	1.84 (0.94–3.60)	0.611	3
90~ (men) or 80~ (women)	2.59 (1.36–4.96)	0.952	4
100~ (men) or 90~ (women)	4.45 (2.28–8.66)	1.493	5
110~ (men) or 100~ (women)	9.69 (4.60–20.42)	2.271	8
History of dyslipidemia			
No	1.00	—	0
Yes	3.18 (2.44–4.15)	1.157	4
Diastolic blood pressure, mmHg			
<70	1.00	—	0
70~	1.97 (1.23–3.16)	0.677	3
80~	2.86 (1.77–4.61)	1.049	4
90~ or treated with anti-hypertensive medication	3.68 (2.32–5.85)	1.304	5
Body mass index, kg/m^2^			
<22	1.00	—	0
22~	1.71 (0.96–3.06)	0.536	2
24~	1.97 (1.17–3.33)	0.679	3
28~	2.58 (1.43–4.65)	0.948	4
30~	4.13 (2.22–7.69)	1.418	5
32~	5.95 (3.12–11.37)	1.784	6

*OR* = odds ratio; *CI* = confidence interval; T2DM = type 2 diabetes mellitus.

**Table 3:** Performance of the RuralDiab risk score and comparison with previously published risk scores for predicting T2DM in validation population.

	Risk variables	AUC	Cutoff value	Number of risk,%	Sensitivity, %	Specificity, %	PPV, %	NPV, %	+LR	−LR
the RuralDiab risk score	Sex, age, family history of diabetes, physical activity, waist circumference, BMI, history of dyslipidemia, DBP	0.723 (0.710–0.735) in total	17	1587 (34.1)	67.9 (61.7–73.6)	67.8 (66.4–69.2)	10.6	97.4	2.11	0.47
		0.711 (0.688–0.732) in men	18	599 (36.5)	70.2 (60.4–78.8)	65.8 (63.3–68.1)	12.2	97.0	2.05	0.45
		0.726 (0.709–0.742) in women	16	1088 (36.1)	70.3 (62.2–77.6)	65.6 (63.8–67.3)	9.4	97.8	2.05	0.45
the New Chinese Diabetes Risk Score	Sex, age, family history of diabetes, waist circumference, BMI, systolic blood pressure	0.708 (0.695–0.721) in total	24	2046 (43.97)	75.5 (69.7–80.7)	57.8 (56.3–59.3)	9.2	97.7	1.79	0.42
		0.686 (0.663–0.709) in men	24	753 (45.9)	74.0 (64.5–82.1)	56.0 (53.5–58.5)	10.2	97.0	1.68	0.46
		0.720 (0.704–0.736) in women	24	1293 (42.9)	76.6 (68.8–83.2)	58.8 (56.9–60.6)	8.6	98.0	1.86	0.40
the ADA score	Age, delivered a macrosomic (≥9 lb) infant, diabetes in parents or siblings, BMI	0.636 (0.622–0.650)^#^ in total	5	1391 (29.9)	49.8 (43.4–56.2)	71.2 (69.9–72.6)	8.9	96.2	1.73	0.70
		0.628 (0.604–0.651)^#^ in men	5	390 (23.8)	44.2 (34.5–54.3)	77.6 (75.5–79.7)	11.8	95.4	1.98	0.72
		0.648 (0.630–0.665)^#^ in women	5	1001 (33.2)	53.8 (45.3–62.1)	67.8 (66.1–69.5)	7.8	96.7	1.67	0.68
the Inter99 score	Sex, age, physical activity, history of diabetes in parent, hypertension, BMI	0.669 (0.655–0.682)^#^ in total	23	1337 (28.7)	53.0 (46.6–59.3)	72.6 (71.3–74.0)	9.9	96.5	1.94	0.65
		0.618 (0.594–0.642)^#^ in men	23	506 (30.8)	49.0 (39.1–59.0)	70.4 (68.0–72.7)	10.1	95.3	1.66	0.72
		0.697 (0.680–0.713) in women	18	1185 (39.3)	69.7 (61.5–77.0)	62.2 (60.4–64.0)	8.5	97.6	1.84	0.49
the Thai risk score	Sex, age, diabetes history of parent or sibling, hypertension, BMI, waist circumference	0.709 (0.695–0.722) in total	6	2109 (45.3)	75.5 (69.7–80.7)	56.4 (54.9–57.9)	8.9	97.6	1.73	0.43
		0.696 (0.673–0.718) in men	7	530 (32.3)	61.5 (51.5–70.9)	69.7 (67.3–72.0)	12.1	96.4	2.03	0.55
		0.713 (0.696–0.729) in women	6	1342 (44.6)	77.9 (70.3–84.4)	57.1 (55.3–59.0)	8.4	98.1	1.82	0.39
the Oman risk score	Age, family history of diabetes, waist circumference, BMI, hypertension	0.675 (0.662–0.689)^#^ in total	10	1646 (35.4)	61.4 (55.1–67.5)	66.1 (64.7–67.5)	9.3	96.8	1.81	0.58
		0.659 (0.636–0.682)^#^ in men	7	692 (42.2)	68.3 (58.4–77.0)	59.6 (57.1–62.1)	10.3	96.5	1.69	0.53
		0.696 (0.679–0.712) in women	10	1284 (42.6)	72.4 (64.4–79.5)	58.8 (56.9–60.6)	8.2	97.7	1.76	0.47

AUC = area under the curve; PPV = positive predictive value; NPV = negative predictive value; +LR = positive likelihood ratio;

−LR = negative likelihood ratio; DBP = diastolic blood pressure; BMI = body mass index; T2DM = type 2 diabetes mellitus.

^#^compared with the RuralDiab risk score *P* < 0.05.

## References

[b1] The International Diabetes Federation. IDF diabetes atlas: Seventh Edition (2015).

[b2] Xu Y. . Prevalence and control of diabetes in Chinese adults. JAMA 310, 948–959 (2013). 24002281 10.1001/jama.2013.168118

[b3] National Diabetes Research Group. A mass survey of diabetes mellitus in a population of 300,000 in 14 provinces and municipalities in China. Chi J Intern Med 20, 678–683 (1981). 7341098

[b4] Lozano R. . Global and regional mortality from 235 causes of death for 20 age groups in 1990 and 2010. Lancet 380, 2095–2128 (2012). 23245604 10.1016/S0140-6736(12)61728-0 PMC10790329

[b5] Murray C. J. . Disability-adjusted life years (DALYs) for 291 diseases and injuries in 21 regions, 1990–2010. Lancet 380, 2197–2223 (2012). 23245608 10.1016/S0140-6736(12)61689-4

[b6] Hussain A., Claussen B., Ramachandran A. & Williams R. Prevention of type 2 diabetes: a review. Diabetes Res Clin Pract 76, 317–326 (2007). 17069920 10.1016/j.diabres.2006.09.020

[b7] American Diabetes Association. Standards of medical care in diabetes- 2011. Diabetes Care 34, S11–S61 (2011). 21193625 10.2337/dc11-S011 PMC3006050

[b8] Kim M. J., Lim N. K., Choi S. J. & Park H. Y. Hypertension is an independent risk factor for type 2 diabetes: the Korean genome and epidemiology study. Hypertens Res 38, 783–789 (2015). 26178151 10.1038/hr.2015.72 PMC4644940

[b9] Sanada H. . High body mass index is an important risk factor for the development of type 2 diabetes. Intern Med 51, 1821–1826 (2012). 22821094 10.2169/internalmedicine.51.7410 PMC3540801

[b10] Rolka D. B. . Performance of recommended screening tests for undiagnosed diabetes and dysglycemia. Diabetes Care 24, 1899–1903 (2001). 11679454 10.2337/diacare.24.11.1899

[b11] Glümer C. . A Danish diabetes risk score for targeted screening: the Inter99 study. Diabetes Care 27, 727–733 (2004). 14988293 10.2337/diacare.27.3.727

[b12] Aekplakorn W. . A risk score for predicting incident diabetes in the Thai population. Diabetes Care 29, 1872–1877 (2006). 16873795 10.2337/dc05-2141

[b13] Al-Lawati J. A. & Tuomilehto J. Diabetes risk score in Oman: a tool to identify prevalent type 2 diabetes among Arabs of the Middle East. Diabetes Res Clin Pract 77, 438–444 (2007). 17306410 10.1016/j.diabres.2007.01.013

[b14] Zhou X. . Nonlaboratory-based risk assessment algorithm for undiagnosed type 2 diabetes developed on a nation-wide diabetes survey. Diabetes Care 36, 3944–3952 (2013). 24144651 10.2337/dc13-0593 PMC3836161

[b15] Wilson P. W. . Prediction of incident diabetes mellitus in middle-aged adults – The Framing-ham Offspring Study. Arch Intern Med 167, 1068–1074 (2007). 17533210 10.1001/archinte.167.10.1068

[b16] Shi L. . Physical Activity, Smoking, and Alcohol Consumption in Association with Incidence of Type 2 Diabetes among Middle-Aged and Elderly Chinese Men. PLoS One 8, e77919 (2013). 24223743 10.1371/journal.pone.0077919 PMC3817165

[b17] Fretts A. M. . Modest Levels of Physical Activity Are Associated With a Lower Incidence of Diabetes in a Population With a High Rate of Obesity: The Strong Heart Family Study. Diabetes Care 35, 1743–1745 (2012). 22723343 10.2337/dc11-2321 PMC3402272

[b18] The InterAct Consortium. Physical activity reduces the risk of incident type 2 diabetes in general and in abdominally lean and obese men and women: the EPIC–InterAct Study. Diabetologia 55, 1944–1952 (2012). 22526603 10.1007/s00125-012-2532-2 PMC3369127

[b19] Qi Q., Liang L., Doria A., Hu F. B. & Qi L. Genetic Predisposition to Dyslipidemia and Type 2 Diabetes Risk in Two Prospective Cohorts. Diabetes 61, 745–752 (2012). 22315312 10.2337/db11-1254 PMC3282815

[b20] Aune D., Norat T., Leitzmann M., Tonstad S. & Vatten L. J. Physical activity and the risk of type 2 diabetes: a systematic review and dose-response meta-analysis. Eur J Epidemiol 30, 529–542 (2015). 26092138 10.1007/s10654-015-0056-z

[b21] Hjellvik V., Sakshaug S. & StrØm H. Body mass index, triglycerides, glucose, and blood pressure as predictors of type 2 diabetes in a middle-aged Norwegian cohort of men and women. Clin Epidemiol 4, 213–224 (2012). 22936857 10.2147/CLEP.S31830 PMC3429151

[b22] World Health Organization. Screening for type 2 diabetes: report of a World Health Organization and International Diabetes Federation Meeting. Geneva: World Health Organization (2003).

[b23] Simmons R. K., Echouffo-Tcheugui J. B. & Griffin S. J. Screening for type 2 diabetes: an update of the evidence. Diabetes Obes Metab 12, 838–844 (2010). 20920035 10.1111/j.1463-1326.2010.01244.x

[b24] China National Center for Food Safety Risk Assessment, Yang, Y. X., Wang, G. Y. & Pan, X. C. *China food composition table, 2nd ed.*, Beijing, China: Peking University Medical Press (2009).

[b25] Chinese Nutrition Society. Chinese Dietary Guidelines (2007). Tibet, China: Tibet People’s Publishing House (2008).

[b26] Craig C. L. . International physical activity questionnaire: 12-country reliability and validity. Med Sci Sports Exerc 35, 1381–1395 (2003). 12900694 10.1249/01.MSS.0000078924.61453.FB

[b27] World Health Organization. Guidelines for controlling and monitoring the tobacco epidemic. (1997)

[b28] Perloff D. . Human blood pressure determination by sphygmomanometry. Circulation 88, 2460–2470 (1993). 8222141 10.1161/01.cir.88.5.2460

[b29] American Diabetes Association: Diagnosis and classification of diabetes mellitus. Diabetes Care 32, S62–S67 (2009). 19118289 10.2337/dc09-S062 PMC2613584

[b30] Pencina M. J., D’Agostino R. B. Sr., D’Agostino R. B. Jr. & Vasan R. S. Evaluating the added predictive ability of a new marker: from area under the ROC curve to reclassification and beyond. Stat Med 27, 157–172 (2008). 17569110 10.1002/sim.2929

[b31] Akobeng A. K. Understanding diagnostic tests 3: Receiver operating characteristic curves. Acta Paediatr 96, 644–647 (2007). 17376185 10.1111/j.1651-2227.2006.00178.x

