# Supplementary material for: Development and evaluation of a risk score for type 2 diabetes mellitus among middle-aged Chinese rural population based on the RuralDiab Study
Source: Sci Rep. 2017 Feb 17;7:42685. doi: 10.1038/srep42685 (PMC5314328; doi:10.1038/srep42685)
Supplement: Supplementary File [file srep42685-s1.pdf]

Title:

Development and evaluation of a risk score for type 2 diabetes mellitus among middle-aged Chinese rural population based on the RuralDiab Study

Running title:

Rural risk score for type 2 diabetes

Authors:

Hao Zhou[1†], Yuqian Li[2†], Xiaotian Liu[1], Fei Xu[1], Linlin Li[1], Kaili Yang[1], Xinling Qian[1], Ruihua Liu[1], Ronghai Bie[1], Chongjian Wang[1*]

Authors affiliations:

[1] Department of Epidemiology and Biostatistics, College of Public Health, Zhengzhou University, Zhengzhou, Henan, *PR* China.

[2] Department of Clinical Pharmacology, School of Pharmaceutical Science, Zhengzhou University, Zhengzhou, Henan, *PR* China.

† Hao Zhou and Yuqian Li contributed equally to this work.

* Correspondence author

Dr. Chongjian Wang and Ronghai Bie

Department of Epidemiology and Biostatistics

College of Public Health, Zhengzhou University

100 Kexue Avenue, Zhengzhou, 450001, Henan, *PR* China

Phone: +86 371 67781452;

Fax: +86 371 67781919

E-mail: tjwcj2005@126.com & bierh2012@126.com

Characteristics	Men(n=1641)	Women(n=3012)	Total(n=4653)	*P*-value
Baseline				
Age(years, mean± SD)	47.15±8.09	45.78±7.91	46.26±8.00	<0.001
Family history of diabetes, n (%)	103 (6.28)	198 (6.57)	301 (6.47)	0.694
High fat intake, n (%)	145 (8.84)	59 (1.96)	204 (4.38)	<0.001
More vegetable and fruit intake, n(%)	768 (46.80)	1172 (38.91)	1940 (41.69)	<0.001
Current smoker, n (%)	990 (60.33)	15 (0.50)	1005 (21.60)	<0.001
Treated with anti-hypertensive medication, n (%)	127 (7.74)	367 (12.18)	494 (10.62)	<0.001
Physical activity, n (%)	1375 (83.79)	2122 (70.45)	3497 (75.16)	<0.001
Waist circumference (cm, mean± SD)	83.85±10.15	82.06±9.74	82.69±9.92	<0.001
Body mass index (kg/m^2, mean± SD)	24.07±3.31	25.04±3.58	24.70±3.52	<0.001
Systolic blood pressure (mmHg, mean± SD)	123.53±16.07	122.73±19.90	123.01±18.65	0.162
Diastolic blood pressure (mmHg, mean± SD)	78.25±10.94	78.75±12.08	78.57±11.69	0.169
Hypertension, n (%)	363 (22.12)	847 (28.12)	1210 (26.00)	<0.001
Dyslipidemia, n (%)	805 (49.06)	1132 (37.58)	1937 (41.63)	<0.001
Fasting glucose (mmol/L, mean± SD)	5.27±0.58	5.30±0.55	5.29±0.56	0.096
Triglycerides (mmol/L, mean± SD)	1.59±1.05	1.60±1.06	1.59±1.06	0.940
HDL-C (mmol/L, mean± SD)	1.12±0.24	1.20±0.26	1.17±0.26	<0.001
Follow-up				
Age (years, mean± SD)	53.30±8.05	51.81±7.89	52.33±7.98	<0.001
Family history of diabetes, n (%)	127 (7.74)	242 (8.03)	369 (7.93)	0.722
High fat intake, n (%)	148 (9.02)	84 (2.79)	232 (4.99)	<0.001
More vegetable and fruit intake, n (%)	349 (21.27)	613 (20.35)	962 (20.67)	0.461
Current smoker, n (%)	863 (52.59)	12 (0.40)	875 (18.81)	<0.001
Treated with anti-hypertensive medication, n (%)	245 (14.93)	567 (18.82)	812 (17.45)	0.001
Physical activity, n (%)	1270 (77.39)	1782 (59.16)	3052 (65.59)	<0.001
Waist circumference (cm, mean± SD)	85.80±10.28	84.52±9.89	84.97±10.05	<0.001
Body mass index (kg/m^2, mean± SD)	24.74±3.48	25.52±3.67	25.25±3.62	<0.001
Systolic blood pressure (mmHg, mean± SD)	124.83±17.22	123.95±19.46	124.26±18.70	0.123
Diastolic blood pressure (mmHg, mean± SD)	79.10±11.15	78.01±11.62	78.39±11.47	0.002
Hypertension (%)	474 (28.88)	963 (31.97)	1437 (30.88)	0.029
Dyslipidemia (%)	1022 (62.28)	1554 (51.59)	2576 (55.36)	<0.001
Fasting blood-glucose (mmol/L, mean± SD)	5.21±1.18	5.11±1.12	5.15±1.14	0.007
Triglycerides (mmol/L, mean± SD)	1.63±1.25	1.61±1.13	1.62±1.17	0.581
HDL-C (mmol/L, mean± SD)	1.03±0.24	1.09±0.26	1.07±0.26	<0.001

Supplementary Table 1. Demographic characteristics of the validation population at baseline and follow-up

SD= standard deviation; HDL-C: high density lipoprotein cholesterol.

Predicted risk (without the addition of risk factors)	Predicted risk (with the addition of risk factors)					% (N) of subjects reclassified with		Net correctly reclassified (%)
	≤2.1%	2.1 to 2.9%	2.9 to 3.9%	3.9 to 5.8%	>5.8%	increased risk	decreased risk	
T2DM patients (N=234)						29.06 (68)	20.09 (47)	8.97
≤2.1%	10	5	5	3	1			
2.1 to 2.9%	6	4	3	3	0			
2.9 to 3.9%	3	10	7	8	11			
3.9 to 5.8%	2	4	9	15	29			
>5.8%	0	1	2	10	83			
Non-diabetes (N=5219)						19.43 (1014)	32.38 (1690)	12.95
≤2.1%	1269	119	95	66	1			
2.1 to 2.9%	246	213	85	57	23			
2.9 to 3.9%	196	359	138	147	113			
3.9 to 5.8%	66	183	305	242	308			
>5.8%	6	14	98	217	653			
Net reclassification improvement								21.92
								Z=4.674 P<0.001

Supplementary Table 2. Reclassification of predicting risk with the addition of risk factors (DBP, BMI and history of dyslipidemia) in T2DM subjects and non-diabetes for the RuralDiab risk score

T2DM= type 2 diabetes mellitus; DBP= diastolic blood pressure; BMI= body mass index.
